# Using multi-criteria decision analysis to describe stakeholder preferences for new quality improvement initiatives that could optimise prescribing in England

**DOI:** 10.3389/frhs.2023.1155523

**Published:** 2023-06-20

**Authors:** Saval Khanal, Kelly Ann Schmidtke, Usman Talat, Alice M. Turner, Ivo Vlaev

**Affiliations:** ^1^ Behavioural Science Group, Warwick Business School, University of Warwick, Coventry, United Kingdom; ^2^ Division of Health Sciences, Warwick Medical School, University of Warwick, Coventry, United Kingdom; ^3^ Liberal Arts, University of Health Sciences and Pharmacy, St Louis, MO, United States; ^4^ Alliance Manchester Business School, University of Manchester, Manchester, United Kingdom; ^5^ Institute for Applied Health Research, University of Birmingham, Birmingham, United Kingdom; ^6^ Heartlands Hospital, University Hospitals Birmingham NHS Foundation Trust, Birmingham, United Kingdom

**Keywords:** multi criteria decision analysis (MCDA), quality improvment, national health service (England), optimising medicine use, decision aid

## Abstract

**Background:**

Hospital decision-makers have limited resources to implement quality improvement projects. To decide which interventions to take forward, trade-offs must be considered that inevitably turn on stakeholder preferences. The multi-criteria decision analysis (MCDA) approach could make this decision process more transparent.

**Method:**

An MCDA was conducted to rank-order four types of interventions that could optimise medication use in England's National Healthcare System (NHS) hospitals, including Computerised Interface, Built Environment, Written Communication, and Face-to-Face Interactions. Initially, a core group of quality improvers (*N* = 10) was convened to determine criteria that could influence which interventions are taken forward according to the Consolidated Framework for Implementation Research. Next, to determine preference weightings, a preference survey was conducted with a diverse group of quality improvers (*N* = 356) according to the Potentially All Pairwise Ranking of All Possible Alternatives method. Then, rank orders of four intervention types were calculated according to models with criteria unweighted and weighted according to participant preferences using an additive function. Uncertainty was estimated by probabilistic sensitivity analysis using 1,000 Monte Carlo Simulation iterations.

**Results:**

The most important criteria influencing what interventions were preferred was whether they addressed “patient needs” (17.6%)' and their financial “cost (11.5%)”. The interventions' total scores (unweighted score out of 30 | weighted out of 100%) were: Computerised Interface (25 | 83.8%), Built Environment (24 | 79.6%), Written Communication (22 | 71.6%), and Face-to-Face (22 | 67.8%). The probabilistic sensitivity analysis revealed that the Computerised Interface would be the most preferred intervention over various degrees of uncertainty.

**Conclusions:**

An MCDA was conducted to rank order intervention types that stand to increase medication optimisation across hospitals in England. The top-ranked intervention type was the Computerised Interface. This finding does not imply Computerised Interface interventions are the most effective interventions but suggests that successfully implementing lower-ranked interventions may require more conversations that acknowledge stakeholder concerns.

## Background

Hospital quality improvement teams, including operational and transformation management teams, are charged with improving patient outcomes and workforce efficacy (1). Often these teams are presented with multiple options for new interventions but have limited resources to implement them all. To determine which interventions to implement trade-offs must be considered that inevitably depend on stakeholder preferences. While many teams already use frameworks such as Six Sigma Lean and The Model for Improvement to organise a scientific process of change (1–5), these frameworks do not help determine which interventions to implement. The multi-criteria decision analysis (MCDA) approach could make this decision-making process more transparent.

MCDA is an umbrella term that describes structured decision-making approaches to prioritise, rank, or choose options based on multiple criteria (6–8). Any MCDA involves three key steps: (a) defining a decision problem that requires choosing among options, (b) selecting multiple criteria upon which those options can be judged, and (c) constructing a performance matrix to rank the options that can serve as a framework for future decisions (9). The MCDA approach is commonly used to help committees, e.g., select job candidates or prioritise new business opportunities (10–12). For narrower decision problems, the MCDA criteria may be equally weighted (i.e., unweighted), as often little information exists to determine the relative importance of each criterion, e.g., to say whether communication or teamwork skills are more important for a particular job. In contrast, for broader decision problems, determining the relative importance (i.e., weights) of each criterion can be a major concern. Here larger samples of diverse stakeholder perspectives can be considered.

MCDA has been used to rank national treatment and reimbursement priorities, and to inform local hospital formularies (8, 9, 13, 14). Formularies are an important component of medication optimisation with cost implications. In England's National Health Service (NHS), the total expenditure on medicines increased by 4.6%, from £16.4 billion in 2019/20 to £17.1 billion in 2020/21. Roughly half (44.4%) of this cost was issued by hospitals (15). NHS hospital trusts already negotiate for the most cost-effective medications and much of this increase is due to the rising patient demand. Interventions dictating which medications clinicians can prescribe could prove effective, but they may be difficult to implement as they appear to question clinician authority or ability to offer patients the best care. Nudge interventions offer an opportunity to optimise medication use without curtailing clinicians' ability to choose the medications they believe best serve patient needs (16).

A recent literature review found 20 nudge interventions issued to optimise medication use in hospital settings of which 16 were successful (9). These nudge interventions are often inexpensive to deliver, e.g., changing message wordings on paper or computerised instructions; however, criteria beyond cost may influence which nudge interventions are the most appealing. The Consolidated Framework for Implementation Research describes 41 empirically and theoretically support criteria that influence the implementation of evidence-based findings (17). Given the large array of nudge intervention options (at least 20) and implementation criteria (at least 41), quality improvement teams could be well served by a framework rank ordering types of nudge interventions they could take forward.

In the current study, an MCDA approach is used to develop a framework that quality improvement teams can use to make more transparent decisions between nudge interventions to optimise medication use in England's NHS hospitals. Additionally, the current study demonstrates how a large number of perspectives can be synthesised to inform a national framework.

## Methods

**Figure 1:**
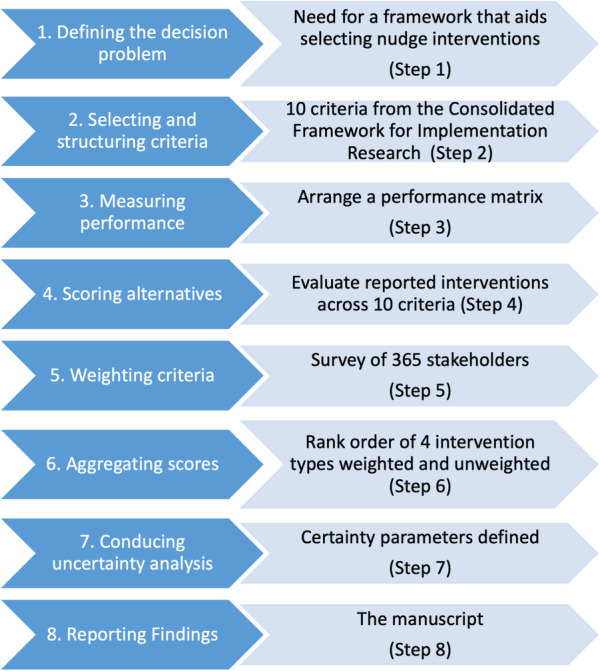
MCDA eight steps and our actions at each step.

We adapted the International Society for Pharmacoeconomics and Outcomes Research (ISPOR) eight-step guidelines for conducting and reporting an MCDA (9). Each step is presented in Figure 1 and detailed further below. The initial four steps describe setting up the problem and the available choice options along with the criteria upon which those options can be judged. In the current project, the first four steps were considered pre-protocol work that shaped our cross-sectional survey. The fifth step involves conducting a cross-sectional survey of national stakeholder preferences for changes within and between criteria. The methods for the first to fifth steps are detailed in the methods section, followed by the sixth and seventh steps in the analysis and the results sections. The final step is to report the findings to the funder and stakeholders.

### Step 1—defining the problem

During early consultations with hospital teams, we presented up to 20 nudge intervention options to optimise medication use previously identified in a systematic review (16). The hospital teams appeared overwhelmed and often did not choose any option, plausibly due to “choice overload” (18). The research team determined that a multi-criteria decision analysis (MCDA) could provide a robust and transparent framework to facilitate these decisions. To further simplify alternative intervention options, the research team categorise the 20 intervention options into a smaller number of nudge intervention types. Our categorisations were cross-checked and approved by representatives at Public Health England.

### Step 2—selecting criteria

To select the criteria upon which the intervention types would be judged, a core group of 10 quality improvers was formed including 4 doctors, 2 pharmacists, and 4 managers. The core group was asked to review the 41 constructs from the Consolidated Framework for Implementation Research (CFIR) (17). Within this framework, constructs are organised across five domains: characteristics of the innovation in question (e.g., two constructs include adaptability and trialability), the individuals involved (e.g., knowledge and self-efficacy), the inner setting (e.g., compatibility and available resources), the outer setting (e.g., incentives and patient needs), and the process encouraging uptake (e.g., planning and opinion leaders). Each improver was asked to rate each construct based on their perceived importance, from 1 (least important) to 5 (most important). Then, each construct was ranked based on the sum of those scores. The top 10 constructs were selected as the criteria considered in the present MCDA.

### Step 3—measuring performance

To measure levels of quality for each criterion, three-point Likert scales were created to indicate high, medium, and low performance levels.

### Step 4—scoring alternatives

Two researchers (SK and UT) used consensus discussions to assess the expected performance of each type of intervention against each criterion. Next, two additional researchers (KAS and IV), each of whom had at least 10 years of experience implementing nudge interventions in healthcare settings, cross-checked these decisions. Next, a performance matrix was assembled with the agreed rankings. A RAG system was used to indicate the quality criteria rankings, such that low-quality rankings would be designated in red, medium in amber, and high in green.

### Step 5—weighting criteria

A cross-sectional survey was conducted between October 2021 and December 2021. The survey was designed using 1,000minds Decision-making software.

#### Participants

To capture more diverse views across a national context, our participants included staff involved in making suggestions or decisions about the implementation of the quality improvement projects for NHS hospital organisations in England such as doctors, nurses, pharmacists, and quality improvement managers (19). As we planned to recruit our participants *via* email, we anticipated a low rate of those emails being opened, 20%–25%, and that only 15%–30% of those opened would be completed (20, 21). We worked with the Health Foundation (described below) to identify at least 4,000 potential participants and anticipated that at least 100 would complete the survey.

#### Identification and recruitment of participants

The opportunity to take part in the survey was initially advertised *via* email to 4,439 members of the Health Foundation's Q-Community. The Health Foundation is a charitable foundation charged with improving healthcare quality. The Q-Community encompasses individuals who self-identify as healthcare quality improvers in England. At the end of the survey, participants were asked to identify other potential participants, i.e., snowball recruitment. The survey was also advertised by researchers on Twitter and LinkedIn. Participants were asked to complete the survey within two weeks. One reminder was sent to encourage completion.

#### Survey

**Figure 2:**
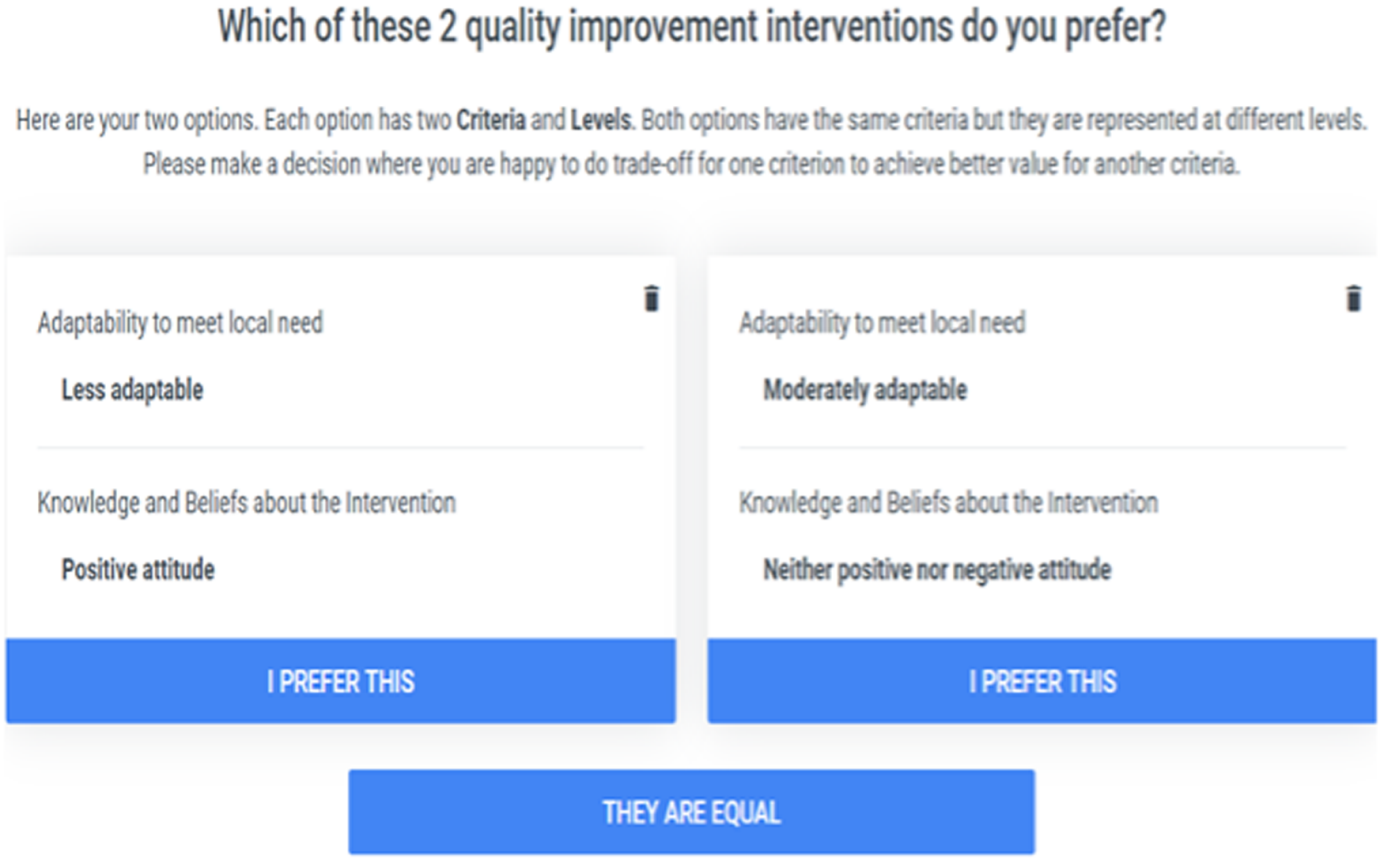
An example of a survey interface for participants.

The survey was designed according to the Potentially All Pairwise Ranking of All Possible Alternatives (PAPRIKA) method (22). PAPRIKA is a method for scoring additive multi-attribute values using pairwise rankings of alternatives (23). Each question asked participants to choose between two alternative intervention types differing according to two criteria with differing performance levels. An example question appears in Figure 2. Future questions were adaptively selected based on participant responses to previous questions.

### Step 6—calculating aggregate scores

Our research team calculated the aggregated scores for each intervention type when the criteria were unweighted and weighted. Unweighted scores were calculated by adding the intervention scores against each criterion based on the researcher team's assigned performance levels. Weighted scores were calculated using an additive model that combines scores and weights in a way that is consistent with stakeholders' expressed preferences. The form of an additive function was given according to the formula

$$V_{j}\;\;=\;\;\overset{n}{\underset{i=1}{\sum }}S_{ij}.\;W_{i},$$

where *V_j_* is the overall value of intervention *j*, *S_ij_* is the score for intervention *j* on criterion *i*, and *W_i_* is the weight attached to criterion *i*. Then, each intervention type was rank ordered according to its total score.

### Step 7—dealing with uncertainty

To assess the consistency of participant responses we developed a decision-analytic model comprising criteria, weights, and scores in TreeAge® Pro R1. A probabilistic sensitivity analysis was carried out using a Monte Carlo simulation with 1,000 iterations for the following three scenarios: (a) incorporating uncertainty in scoring the alternatives, (b) incorporating uncertainty in the weighting preference, and (c) incorporating uncertainty for both the scoring alternatives and weighing preference. A gamma distribution was used for scores. Because preference weights are positive proportions that totalled one, we selected a Dirichlet distribution to preference weights in the simulation (24). The means of observed data were used as parameters for this distribution.

### Step 8—reporting and examining findings

After the final report is shared with the Health Foundation, a brief synopsis of the results will be made available to members of the Q-Community along with a link to the publication for further information.

## Results

### Step 1—agreed decision problem

The research team determined that a multi-criteria decision analysis (MCDA) could help quality improvement teams select nudge interventions to optimise medication use. To promote a more generalisable framework, the 20 nudge interventions located in the systematic review were grouped into 4 intervention types, including Computerised Interfaces; Built Environment; Written Communications; and Face-to-Face Interactions. Computerised Interface interventions could include reminder prompts or default choice orders presented on the computer. Built Environment interventions could involve changing the physical space within which care occurs or the materials available. Written Communication interventions could include letters or emails to staff or adjustments to existing paper forms. Face-to-Face Interaction interventions could involve meetings within and across departments.

### Step 2—selected criteria

The 10 selected decision criteria are provided in Table 1 in the order the working group advised. All of the Consolidated Framework for Intervention Research's (CFIR's) domains were represented among the selected criteria, including four intervention criteria, two outer setting criteria, two individual characteristics criteria, one inner setting criterion, and one implementation process criterion. Our working group advised that the original terms used in the CFIR were too technical for our intended participants. Informed by each construct's definition, KAS and IV simplified the terms by adding implementation context. In the second column of Table 1, added words are given in italics.

**Table 1:** The 10 criteria considered, the domain's definitions, and measurement levels.

CFIR Domain	Criteria	Definition	Levels
Outer Setting	Patient needs and resources	The extent to which patient needs, as well as barriers and facilitators to meet those needs, are accurately known and prioritised by the organisation	Highly addresses patient needs Moderately addresses patient needs Does not address patient needs
Intervention Characteristics	Cost *to implement*	Costs of the intervention and costs associated with implementing the intervention including investment, supply, and opportunity costs	Cheap Neither expensive nor cheap Expensive
Intervention Characteristics	Adaptability *to meet local needs*	The degree to which an intervention can be adapted, tailored, refined, or reinvented to meet local needs	Highly adaptable Moderately adaptable Less adaptable
Inner Setting	Relative priority *of implementing intervention*	Individuals’ shared perception of the importance of the implementation within the organisation	High priority Medium priority Low priority
Intervention Characteristics	Relative advantage *of implementing intervention*	Stakeholders’ perception of the advantage of implementing the intervention vs. an alternative solution	High advantage Medium advantage Low advantage
Inner setting	Available resources *for implementation*	The level of resources dedicated for implementation and ongoing operations, including money, training, education, physical space, and time	Highly available Moderately available Less available
Intervention Characteristics	Evidence *about the intervention's impact*	Stakeholders’ perceptions of the quality and validity of evidence supporting the belief that the intervention will have desired outcomes	Strong evidence Moderate evidence Low evidence
Characteristics of Individuals	Knowledge and beliefs about intervention	Individuals’ attitudes toward and the value placed on the intervention as well as familiarity with facts, truths, and principles related to the intervention	Positive attitude Neither positive nor negative attitude Negative attitude
Characteristics of Individuals	Identification with organisation	A broad construct related to how individuals perceive the organisation, and their relationship and degree of commitment with that organisation	Highly known in the organisation Moderately known in the organisation Not well known in the organisation
Implementation Process	Planning to *facilitate the intervention implementation*	The degree to which a scheme or method of behaviour and tasks for implementing an intervention are developed in advance, and the quality of those schemes or methods	Well planned Moderately planned Not well planned

### Step 3—measured performance

The measures of performance assigned are stated in the final column of Table 1. For example, the CFIR criterion “adaptability to meet local needs” was measured by using each of the following three values: 1 = Highly adaptable, 2 = Moderately adaptable, or 3 = Less adaptable.

### Step 4—alternatives scored

The scores for each intervention type by criteria were assembled in a performance matrix (Table 2). Only the Written Communications and Face-to-Face Interactions intervention types had low-quality ranking aspects.

**Table 2:** Performance matrix.

Criteria	Computerised interface	Built environment	Written communication	Face-to-face
Patient needs and resources	Moderately addresses patient needs	Moderately addresses patient needs	Moderately addresses patient needs	Moderately addresses patient needs
Cost to implement	Cheap	Cheap	Cheap	Expensive
Adaptability to meet local needs	Highly adaptable	Highly adaptable	Highly adaptable	Moderately adaptable
Relative priority of implementing intervention	Medium priority	Medium priority	Medium priority	Medium priority
Relative advantage of implementing intervention	Moderately advantage	Moderately advantage	Low advantage	Highly advantage
Available resources for implementation	Highly available	Highly available	Moderately available	Less available
Evidence about the intervention's impact	Moderate evidence	Moderate evidence	Moderate evidence	High evidence
Knowledge and beliefs about intervention	Positive attitude	Positive attitude	Positive attitude	Positive attitude
Identification with organisation	Moderately known	Moderately known	Moderately known	Highly known
Planning to facilitate the intervention implementation	Well planned	Well planned	Moderately planned	Moderately planned

### Step 5—weighted criteria

#### Participants

The survey was completed by 356 participants, which was greater than anticipated. The average participant completed the survey in 8.6 (SD = 2.3) minutes. Most participants identified as male (57%), were between 31 and 50 years old (76.4%), and had 6 to 10 years of professional work experience (40%). All nine geographic regions of England were represented. Further details are presented in Table 3.

**Table 3:** Demographic characteristics of the participants.

Demographics	Total number (*N*)	Percentage (%)^a^
Gender		
Male	202	56.7%
Female	143	40.2%
Non-binary	7	2.0%
Do not want to disclose	4	1.1%
Age in years		
Less than 30 years	30	8.4%
31- 40 year	163	45.8%
41–50 years	109	30.6%
51–60 years	41	11.5%
More than 60 years	9	2.5%
Do not want to disclose	4	1.1%
Length of experience		
Less than 1 year	9	2.5%
1–5 years	106	29.8%
6–10 years	147	41.3%
11–15 years	49	13.8%
More than 15 years	45	12.6%
Job Title^b^		
Quality Improvement Officer/ Manager	113	31.7%
Doctor (Hospital)	63	17.7%
Nurse	54	15.2%
General Practitioner	52	14.6%
Pharmacist	49	13.8%
Hospital Administrator	45	12.6%
Others	17	4.8%
CCG Member	13	3.7%
Location		
East of England	55	15.4%
East Midlands	45	12.6%
North West	45	12.6%
South West	45	12.6%
South East	41	11.5%
Yorkshire and the Humber	36	10.1%
West Midlands	36	10.1%
Greater London	32	9.0%
North East	21	5.9%

^a^ Percentages may not sum to 100% due to rounding.

^b^ Participants were allowed to select multiple job titles.

#### Preference weight for criteria

**Figure 3:**
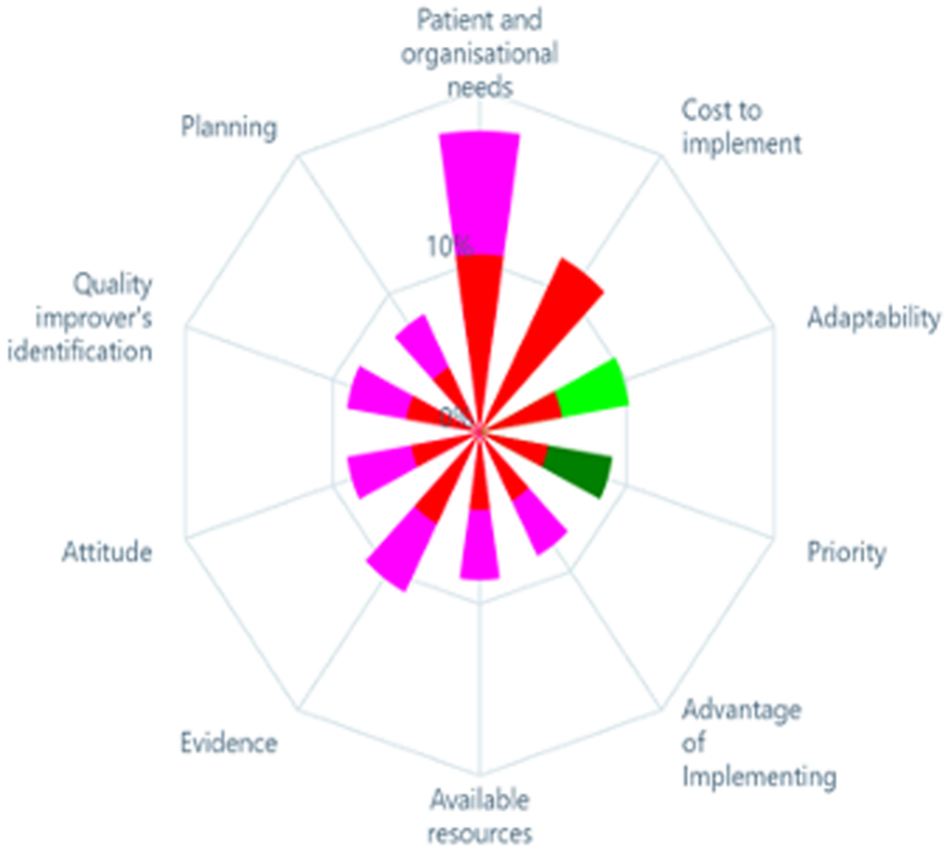
Polar chart of the criterion weights.

The weights given by the participants to each criterion are represented in Figure 3. Based on the mean preference values, the most highly weighted criterion appears at the top of the chart, followed, in a clockwise direction, by the second-most highly weighted criterion, and so on. Based on the preference of stakeholders, whether a particular intervention addresses patient needs was the most important (17.6% weight out of 100% was attributed to this criteria) followed by its likely financial cost (11.5%), and evidence supporting its effectiveness (10.5%). Planning required to facilitate the intervention implementation (7.8%) was the least important criterion. In addition to the criteria weights, the weights for each level within the criteria were also calculated, see Table 4.

**Table 4:** Weights for each criterion and level.

Criteria and levels	Weight of criteria and level (in percentage)
**Patient needs**	—
Does not address patient and organisational needs	0%
Moderately address a patient and organisational need	10.4%
Highly addresses patient and organisational needs	17.6%
**Cost to implement**	—
Expensive	0%
Neither expensive nor cheap	6.2%
Cheap	11.5%
**Evidence**	—
Low evidence	0%
Medium evidence	6.0%
High evidence	10.5%
**Adaptability**	—
Less adaptable	0%
Moderately adaptable	5.4%
Highly adaptable	9.8%
**Knowledge and beliefs about intervention**	—
Negative attitude	0%
Neither positive nor negative attitude	4.5%
Positive attitude	8.7%
Identification with organisation	—
Not well known in the organisation	0%
Moderately known in the organisation	4.8%
Well-known in the organisation	8.7%
Relative priority	—
Low priority	0%
Medium priority	4.5%
High priority	8.7%
**Available resources**	—
Less resources	0%
Medium resources	4.5%
High resources	8.6%
**Relative advantage of implementing**	—
Low advantage	0%
Medium advantage	4.5%
High advantage	8.1%
**Planning to facilitate the intervention**	—
Not well planned	0%
Moderately planned	4.3%
Well planned	7.8%

Each sector in the chart represents a criterion and the preference values for each of its levels.

### Step 6 –Aggregate scores

Table 5 displays the ranking of different intervention types based on their total scores in two scenarios: unweighted and weighted. In the unweighted scenario, the Computerised Interface intervention type garnered the highest total score of 25, closely followed by the Built Environment with a score of 24. Both the Written Communication and Face-to-Face intervention types received a total score of 22, jointly ranking third. In the weighted scenario, the top two intervention types remain the same as in the unweighted scenario, with Computerised Interface receiving a total score of 83.8 and the Built Environment receiving 79.6. However, the ranking of Written Communication and Face-to-Face interventions change. Written Communication receives a total score of 71.6, ranking third, while Face-to-Face interaction receives a total score of 67.8, ranking fourth.

**Table 5:** Scoring and ranking of the interventions.

Unweighted scores	Computerised interface	Built environment	Written communication	Face-to-face
Patient needs and resources	2	2	2	2
Cost to implement	3	3	3	1
Adaptability to meet local needs	3	3	3	2
Relative priority of implementing intervention	2	2	2	2
Relative advantage of implementing intervention	2	2	1	3
Available resources for implementation	3	2	2	1
Evidence about the intervention's impact	2	2	2	3
Knowledge and beliefs about intervention	3	3	3	3
Identification with organisation	2	2	2	3
Planning to facilitate the Intervention Implementation	3	3	2	2
Total score	25	24	22	22
Rank	First	Second	Joint third	Joint third
**Weighted scores**				
Patient needs and resources	17.6	17.6	17.6	17.6
Cost to implement	11.5	11.5	11.5	0
Adaptability to meet local needs	9.8	9.8	9.8	5.4
Relative priority of implementing intervention	4.5	4.5	4.5	4.5
Relative advantage of implementing intervention	4.5	4.5	0	8.1
Available resources for implementation	8.6	4.4	4.4	0
Evidence about the intervention's impact	6	6	6	10.5
Knowledge and beliefs about intervention	8.7	8.7	8.7	8.7
Identification with organisation	4.8	4.8	4.8	8.7
Planning to facilitate the Intervention Implementation	7.8	7.8	4.3	4.3
Total score	83.8	79.6	71.6	67.8
Rank	First	Second	Third	Fourth

These scores represent the preferences given by quality improvers in the NHS to different criteria, including patient needs and resources, cost to implement, adaptability to meet local needs, relative priority and advantage of implementing the intervention, available resources for implementation, evidence about the intervention's impact, knowledge and beliefs about the intervention, identification with the organisation, and planning to facilitate intervention implementation. The weights for these criteria were obtained from the perceived importance or preference assigned by the quality improvers in the NHS. The weighted scores are given as percentages, reflecting the importance assigned to each criterion.

### Step 7—capturing uncertainty

**Figure 4:**
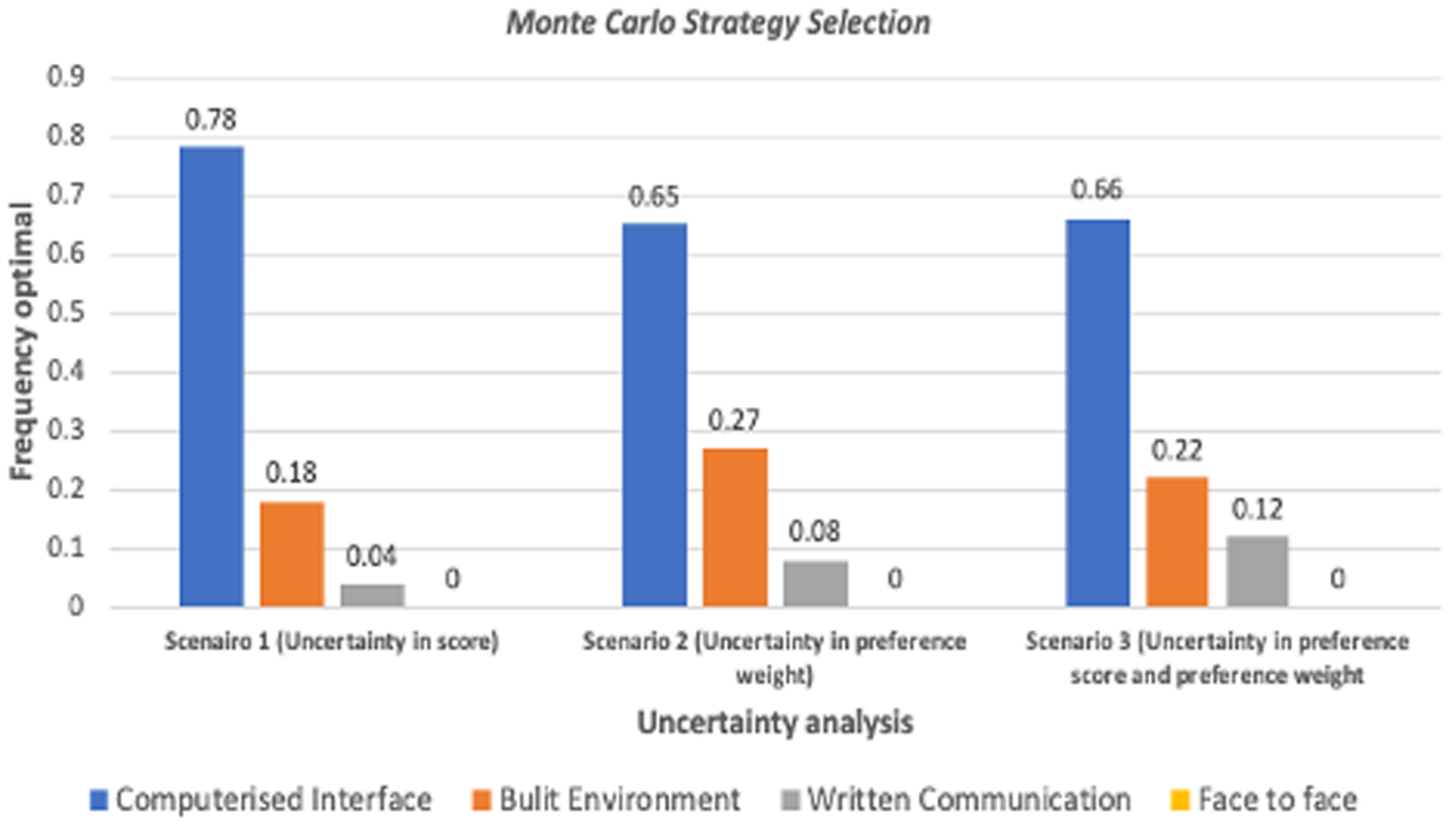
Preference for intervention types over three scenarios.

Table 6 and Figure 4 illustrate the outcomes of a Monte Carlo simulation (probabilistic sensitivity analysis), presenting the anticipated scores for distinct intervention types across various scenarios. The scenarios comprise the base model (expected scores), uncertainty in scoring, uncertainty in weighting preferences, and uncertainty in both scoring and weighting. In all scenarios, the Computerised Interface intervention type remains the top-ranked intervention, receiving the highest expected scores. For instance, in scenario 1 (uncertainty in scoring), the anticipated values for Computerised Interface interventions indicate that they are likely to be chosen 78 times out of 100. Conversely, the Face-to-Face intervention type does not receive the highest expected scores in any of the scenarios, indicating that it is less probable to be prioritised over the other three types.

**Table 6:** Effects of uncertainty in the weighted scores (expected values) of the project.

	Computerised interface	Built environment	Written communication	Face-to-face
Base model, Expected Values (Mean score, 95% CI)	83.8 (74.2, 89.3)	79.6 (70.2, 87.0)	71.6 (61.5, 81.7)	67.8 (58.9, 77.7)
Scenario 1 (uncertainty in scoring) Expected Values (Mean score, 95% CI)	80.7 (77.9, 83.2)	76.5 (74.8, 80.1)	69.0 (67.5, 70.5)	66.8 (56.6, 69.6)
Scenario 2 (uncertainty in weighing preference) Expected Values (Mean score, 95% CI)	83.6 (76.7, 85.6)	79.0 (70.3, 85.2)	71.6 (64.1, 79.5)	67.1% (60.3, 74.5)
Scenario 3 (uncertainty in scoring and weighing preference) Expected Values, (Mean score, 95% CI)	80.7 (73.4, 86.5)	76.4 (69.9, 82.2)	69.0 (62.6, 75.5)	66.9 (60.6%, 73.1)

The anticipated values for each intervention type are presented in percentage, with their mean scores and 95% confidence intervals. In the base model, the expected scores for Computerised Interface, Built Environment, Written Communication, and Face-to-Face interventions are 83.8, 79.6, 71.6, and 67.8, respectively. In scenario 1, the anticipated scores decrease slightly for all intervention types, but the rank order stays the same.

In scenario 2 (uncertainty in weighting preferences), the anticipated scores for all intervention types are comparable to those in the base model, signifying that the ranking of the intervention types is not substantially affected by uncertainty in weighting preferences. In scenario 3 (uncertainty in scoring and weighting preferences), the expected scores decrease further for all intervention types, but the rank order remains the same as in the base model. Overall, the outcomes suggest that the Computerised Interface intervention type is the most highly prioritised intervention for quality improvement in healthcare settings, with the Built Environment intervention type ranking second.

## Discussion

A multicriteria decision analysis (MCDA) was conducted to describe quality improvers' preference for implementing nudge-type interventions to optimise medication use for NHS hospitals in England. Our findings suggest that quality improvers' intervention preferences are most influenced by patient needs followed by the interventions' financial costs. While the top-ranked intervention types, Computerised Interface interventions and Built Environment interventions, were the same for weighted and unweighted scores, preferences for the bottom-ranked interventions were different. Specifically, while the research team's unweighted rankings predicted that the Written Communications and Face-to-Face interventions would not differ, quality improvers preferred Written Communication interventions over Face-to-Face interventions. Our resultant framework could serve as a decision aid to assist emerging quality improvement initiatives across NHS hospitals in England.

MCDA can be a useful tool for decision-making in quality improvement programmes. Based on the authors' knowledge, this is the first paper in which MCDA has been performed for a quality improvement programme. But the MCDA approach is not entirely new. For example, MCDA has been used to prioritise non-pharmacological treatment options for patients with abdominal cancer suffering from chronic pain (25). In that study, a smaller number of health workers than in the current study, just five clinicians, rated each treatment across five criteria including convenience, pain, risk, duration, and cost. Convenience was weighted higher than costs, and similar to our findings their findings differed depending on whether the criteria were weighted. In another study (26), MCDA was used to prioritise different groups of people who would be offered COVID-19 vaccinations when vaccines were in short supply. Here, 60 physicians took part. Note that in both cases, participants were limited to health workers.

Outside of treatment-focused decisions, MCDA has been used to prioritise funding for a national healthcare service in New Zealand (27). Here, focus group discussions took place involving diverse stakeholder groups: 4 members of the public, 10 front-line workers, 7 retirees, 5 public health workers, and 13 members of a health provider group focused on an indigenous population. Stakeholder discussions revolved around not just how to score alternatives, but also around what the criteria meant. How and when to bring different stakeholder groups into the discussion making processes is an important consideration. While considering divergent perspectives may increase buy-in across stakeholder groups, it will also take considerable time and resources to foster a shared understanding of the criteria assessed and the quantitative processes underlying the resultant scores (14).

The present study focuses on the preferences of quality improvers, as this stakeholder group is most directly charged with building and sustaining the momentum necessary for successful implementation. Quality improvers' preferences were assessed across ten of the Consolidated Framework for Implementation Researcher (CFIR) constructs, as this is a widely recognized framework to support implementation. The CFIR constructs include information that quality improvers can meaningfully react to in a self-completion online survey; these constructs could prove challenging for other stakeholder groups to meaningfully react to in this setting. Of course, to build and sustain momentum, quality improvers must understand the preferences of other stakeholders, including patients whose voices should be heard as the intervention takes shape. The MCDA process could be used to foster shared decision-making and understanding across stakeholder groups and determine how a quality improvement programme is implemented, e.g., when, what materials, how often, etc.”

Overall, MCDA can be a versatile tool in healthcare decision-making, from evaluating treatment options, to prioritising project funding and quality improvement programmes.

### Limitations

A limitation of the MCDA approach is that the ultimate framework will depend on the participants who take part (28). While our participant sample is large, it is a convenience sample, and we did not collect sufficient demographics to ensure historically underrepresented groups were represented. Further, it is uncertain whether the preferences captured in this survey will remain stable as interventions themselves evolve. That said, as the capabilities of computerised software itself and the people who use that software expand, we suspect that preferences for computerised interventions are likely to remain stable or increase (29, 30).

Another limitation of the MCDA approach is that it only reveals which intervention options people prefer; it does not tell us which intervention options are most likely to be effective. The authors acknowledge that any decision to initiate or implement quality improvement programmes in the NHS would be a multifactorial decision, however, understanding quality improvers' preferences is a key issue. For example, many interventions that have the potential to be effective in optimal conditions fail to be effective where workers do not support their use. Thus, where a less preferred intervention type is rolled out, extra attention in the implementation process should be devoted to increasing people's acceptance (31).

### Implications

One implication of our finding is that while the top preferred intervention options may be clear, the ordering of lower-ranked interventions may be less certain. When the top-ranked option is not feasible, researchers should work closely with stakeholders to select and co-design the implementation process for less preferred intervention options. This recommendation likely extends to complex interventions, which may include combinations of high-ranked and lower-ranked intervention types (32). For instance, a computerised decision aid integrated into the hospital prescribing software may include prompts facilitating shared decision-making with pharmacists, patients, and carers. Where these prompts minimise perceived threats to professional autonomy and preserve a natural doctor-patient conversation, they are more likely to be accepted (33). Implementing such an intervention may require face-to-face meetings in advance to co-design an acceptable prompt.

The current study demonstrates how many perspectives can be synthesised to inform a national framework. Implementing such a project required access to quality improvers across England along with statistical expertise and dedicated software. As new decisions arise within local settings, a narrower MCDA may suffice to transparently capture stakeholder perspectives and to consider a broader range of criteria (7). Where possible, patients could be part of this process (34). Wider implementation of the MCDA methodology may require further top-down support and bottom-up capacity (35). For instance, demand issues may stem from board members being unaware that these decisions could be more transparent. Supply issues may stem from quality improvers not knowing how to gather or present this type of support. Multi-tiered initiatives could be used to simultaneously address these supply and demand issues (36).

## Conclusion

A multicriteria decision analysis framework revealed that quality improvers prefer medication-optimising interventions that address patient needs and that are less financially costly. Thus, where the costs of two nudge interventions are similar, preference will largely turn on the perceived ability of the intervention to meet patient needs. At the time of this study, the Computerised Interface intervention type was most preferred. This finding aligns with expanding technological capabilities in healthcare and may promote future innovative low-cost opportunities to overcome practical problems. The current research team cautions that stakeholders do not always prefer what is likely to be the most effective intervention. Interventions that have the potential to be effective but are not immediately preferred must be implemented in a manner that respects stakeholders' preferences.

## Data Availability

The original contributions presented in the study are included in the article, further inquiries can be directed to the corresponding authors.
